# Predicting Breast Cancer in Breast Imaging Reporting and Data System (BI-RADS) Ultrasound Category 4 or 5 Lesions: A Nomogram Combining Radiomics and BI-RADS

**DOI:** 10.1038/s41598-019-48488-4

**Published:** 2019-08-15

**Authors:** Wei-quan Luo, Qing-xiu Huang, Xiao-wen Huang, Hang-tong Hu, Fu-qiang Zeng, Wei Wang

**Affiliations:** 1 Department of Ultrasonography, Zhongshan Hospital of Traditional Chinese Medicine, Affiliated to Guangzhou University of Chinese Medicine, Zhongshan, People’s Republic of China; 2 Department of Nephrology, Zhongshan Hospital of Traditional Chinese Medicine, Affiliated to Guangzhou University of Chinese Medicine, Zhongshan, People’s Republic of China; 3 Department of Medical Ultrasonics, Institute of Diagnostic and Interventional Ultrasound, The First Affiliated Hospital of Sun Yat-sen University, Guangzhou, People’s Republic of China

**Keywords:** Breast cancer, Breast cancer

## Abstract

Radiomics reflects the texture and morphological features of tumours by quantitatively analysing the grey values of medical images. We aim to develop a nomogram incorporating radiomics and the Breast Imaging Reporting and Data System (BI-RADS) for predicting breast cancer in BI-RADS ultrasound (US) category 4 or 5 lesions. From January 2017 to August 2018, a total of 315 pathologically proven breast lesions were included. Patients from the study population were divided into a training group (n = 211) and a validation group (n = 104) according to a cut-off date of March 1^st^, 2018. Each lesion was assigned a category (4A, 4B, 4C or 5) according to the second edition of the American College of Radiology (ACR) BI-RADS US. A radiomics score was generated from the US image. A nomogram was developed based on the results of multivariate regression analysis from the training group. Discrimination, calibration and clinical usefulness of the nomogram for predicting breast cancer were assessed in the validation group. The radiomics score included 9 selected radiomics features. The radiomics score and BI-RADS category were independently associated with breast malignancy. The nomogram incorporating the radiomics score and BI-RADS category showed better discrimination (area under the receiver operating characteristic curve [AUC]: 0.928; 95% confidence interval [CI]: 0.876, 0.980) between malignant and benign lesions than either the radiomics score (*P* = 0.029) or BI-RADS category (*P* = 0.011). The nomogram demonstrated good calibration and clinical usefulness. In conclusion, the nomogram combining the radiomics score and BI-RADS category is potentially useful for predicting breast malignancy in BI-RADS US category 4 or 5 lesions.

## Introduction

Conventional ultrasound (US) is an essential imaging technique for the detection or diagnosis of breast lesions. Breast US has been widely used for differentiating between malignant and benign lesions^1,2^. In 2003, the American College of Radiology (ACR) standardized diagnostic characterization of ultrasound-detected breast lesions in the fourth edition of the Breast Imaging Reporting and Data System (BI-RADS®) atlas (first edition of the ACR BI-RADS US)^3^. After a decade of clinical practice, the ACR updated the BI-RADS US in 2013 (second edition of the ACR BI-RADS US)^4^.

In the second edition of the ACR BI-RADS US atlas, breast lesions are ultimately assigned a category after analysing their sonographic features^4^. There are seven categories in total^4^. Category 0 is defined as a diagnosis that needs to be combined with other imaging. Category 1 is defined as no lesions or negative findings. Category 2 is defined as benign lesion without suspicious characteristics. Category 3 is defined as benign possible with less than 2% malignant probability. Category 4 is defined as suspicious lesion with 2% to 95% malignant probability that is recommended for biopsy. Category 5 is defined as highly suspected of malignancy, with more than 95% malignant probability. Category 6 is defined as known malignancy or pathologically proven to be malignant. Because of the wide range of malignance probability, category 4 is divided into three subcategories: 4A, 4B and 4C, with 2–10%, 10–50% and 50–95% malignance probability, respectively^4^.

However, sonographic features for determining BI-RADS categories are generally based on the radiologist’s interpretation. In addition, microcosmic features of images, such as texture features, may not be identified by visual interpretation. Radiomics is a novel computer-aided technology that reflects the texture and morphological features of tumours by quantitatively analysing the grey values of medical images^5–8^. Radiomics can extract many quantitative features from medical images through a computer algorithm^9–11^. Most of the quantitative features extracted through computerized algorithms are beyond visual interpretation but may potentially be associated with important clinical outcomes^9,10,12^. Therefore, we hypothesized that these potential quantitative features extracted from US images could predict the malignancy of breast lesions.

We aimed to develop a radiomics score from the breast US images. Then, a nomogram incorporating the radiomics score and BI-RADS category was developed to predict the malignancy of breast lesions. We focused our study on breast lesions classified as ACR BI-RADS US categories 4 or 5 because these lesions have a wide-ranging likelihood of malignancy (>2%) and were recommended for biopsy.

## Methods

### Study population

The study was approved by the review board of Guangzhou University of Chinese Medicine and complied with the *Declaration of Helsinki*. Informed consent was waived because the present study is retrospective. From January 2017 to August 2018, female patients with US findings of breast lesions were continuously collected and were further selected according to the following inclusion and exclusion criteria.

The inclusion criteria were as follows: (1) a pathological result was available; (2) breast US was performed before biopsy or resection; (3) US examination was performed using an Aplio 500 (Toshiba Medical Systems, Tokyo, Japan) equipped with a PLT-1005BT linear array probe; and (4) the target lesion was assigned as BI-RADS category 4A, 4B, 4C or 5 according to the second edition of the ACR BI-RADS US atlas.

The exclusion criteria were as follows: (1) the pathological result was indefinite; (2) the patient had undergone anticancer therapy (radiotherapy or chemotherapy); or (3) the target lesion was incompletely visible on US.

**Figure 1:**
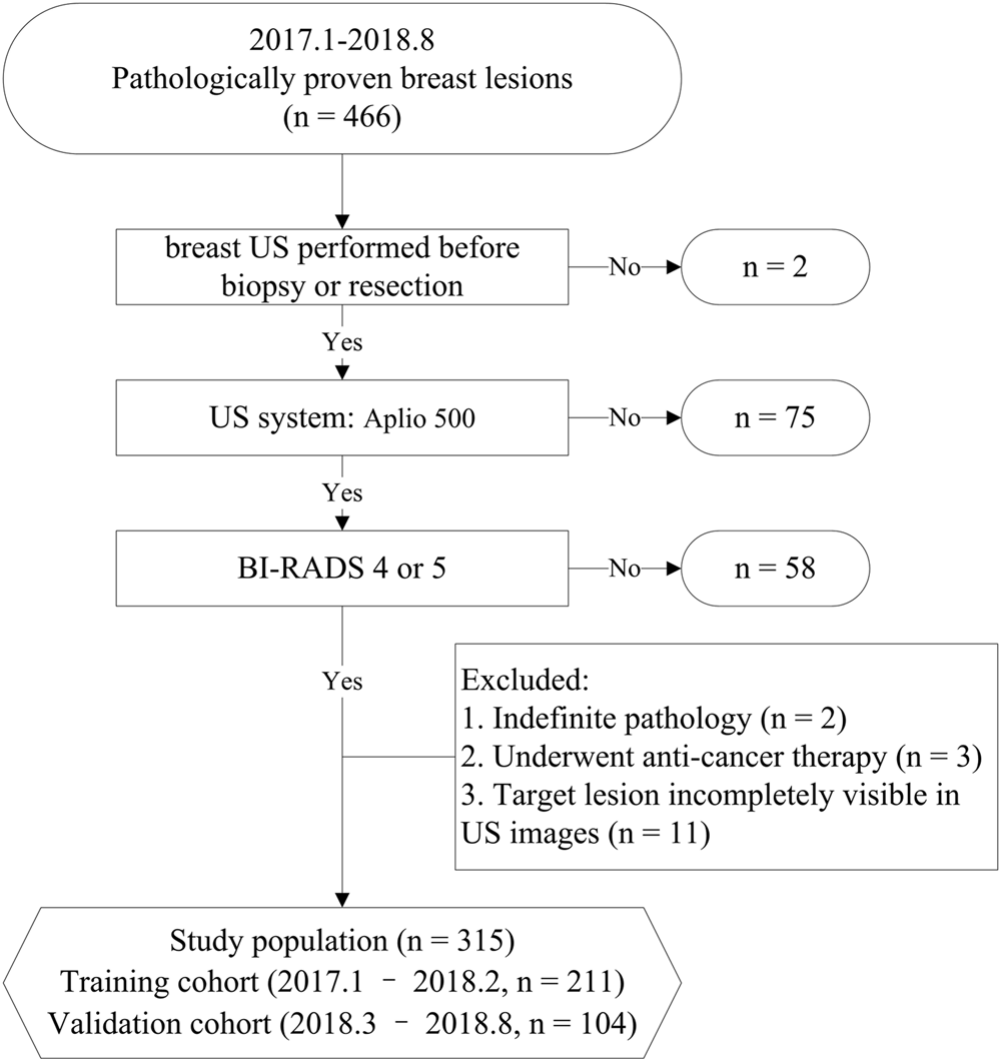
Flow chart of the study population enrolment.

For patients with more than one lesion that was BI-RADS category 4A or higher, only the lesion with the highest BI-RADS category was included in the analysis to guarantee the statistical independence of each observation. Finally, a total of 315 lesions from 315 women (mean age, 44.9 ± 8.6 years; range, 24 to 83 years) were included (Fig. 1). Patients evaluated between January 2017 and February 2018 were included as the training group (211 patients; mean age, 44.1 ± 7.6 years; range, 24 to 69 years), and those evaluated between March 2018 and August 2018 were included as the validation group (104 patients; mean age, 46.7 ± 10.1 years; range, 25 to 83 years).

### US and pathological examinations

US examinations were performed using an Aplio 500 (Toshiba Medical Systems, Tokyo, Japan) equipped with a PLT-1005BT linear array probe. All of the lesions were examined and assessed by the same radiologist (**W.L**.) with over 10 years of experience of breast US examination. Imaging parameters were adjusted to optimally visualize the target lesion. The greyscale image of the target lesion with the largest long axis cross-section was routinely stored on the hard disk. Additional images containing important features (colour flow, calcification, halo, etc.) were also stored. The largest diameter of each lesion was recorded. Each lesion was described as complying with the second edition of the ACR BI-RADS US atlas and was ultimately assigned a category (BI-RADS 4A, 4B, 4C or 5)^4^. The radiologist was not blinded to the patients’ clinical characteristics.

In our practice, lesions classified as BI-RADS category 4A or higher were all recommended for biopsy. Pathological results were confirmed by US-guided biopsy or surgery. US-guided biopsy was performed using a core instrument with a 14-gauge needle or a vacuum-assisted biopsy machine with an 8-gauge needle. More than three tissue samples were obtained and placed in formalin solution and then processed for histopathology by standard procedures^13^. Patients with indefinite histological results were recommended for surgery.

### Radiomics score

**Figure 2:**
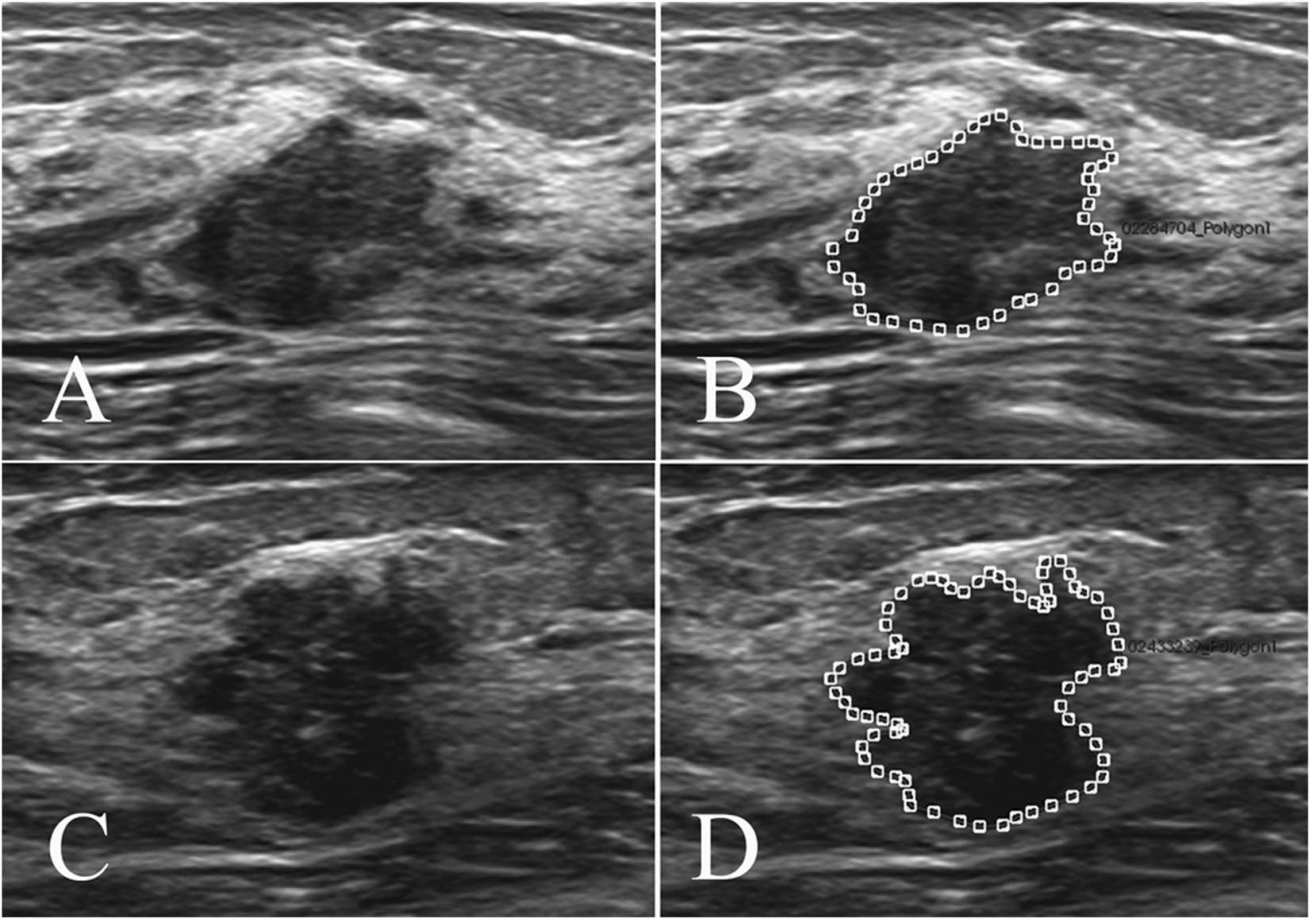
Examples of delineating regions of interest (ROIs) on US images. The greyscale image (**A**) and the ROI (**B**) of a benign lesion with the largest long-axis cross-section (radiomics score = −1.016). The greyscale image (**C**) and the ROI (**D**) of a malignant lesion with the largest long-axis cross-section (radiomics score = −0.791).

A radiomics score was calculated for each lesion with radiomics techniques, which were reported in our previous study^14^. First, the greyscale US images with the largest long axis cross-section of all target lesions were exported from the US machine and imported into the A.K. software (Artificial Intelligence Kit, version 1.1, GE Healthcare, Little Chalfont, UK). Then, the radiologist (**W.L**., who performed the US examination) delineated the margin of each target lesion as the region of interest (ROI) using A.K. software (Fig. 2).

Discretization of the grey values was performed using a fixed bin size. In the A.K. software, the parameter of the bin size is the binwidth, which was set to 25 by default. After delineating the ROI, the software automatically extracted radiomics features while completing the discretization step.

A total of 1,044 radiomics features were extracted from each ROI by the A.K. software. Least absolute shrinkage and selection operator (LASSO) regression was used to select significant features^15^. Then, a formula incorporating the selected features was developed to calculate the radiomics score. More details of the formula development process are presented in the Additional file (Appendix A1).

To assess the intra-observer reproducibility, the radiologist (**W.L**.) performed the second extraction of radiomics features from 50 randomly chosen images after 1 week according to the same procedure. Intra-class correlation coefficient (ICC) was used to assess the intra-observer agreement, which was graded as very good (0.80 to 1.00), good (0.60 to 0.80), fair (0.40 to 0.60), moderate (0.20 to 0.40) or poor (<0.20).

### Development of the nomogram

A nomogram for predicting breast malignancy was developed using data from the training group. Univariate and multivariate logistic regression analyses were performed to analyse the significant factors associated with breast malignancy. Candidate factors included age, largest lesion diameter, BI-RADS category and radiomics score. In univariate analysis, factors with *P* values less than 0.10 were included in the multivariate analysis. Then, factors with *P* values less than 0.05 were considered independent predictors after the multivariate analysis. Finally, a nomogram was developed by incorporating these independent predictors.

### Validation of the nomogram

The performance of the nomogram for predicting breast malignancy with respect to discrimination, calibration, and clinical usefulness was evaluated with the validation group.

#### Discrimination

Receiver operating characteristic (ROC) curves were plotted to assess the performance of the nomogram for discriminating malignant from benign lesions in the training and validation groups. Discrimination was quantified with the area under the ROC curve (AUC). The optimal cut-off value of the radiomics score that was calculated from the training group was applied in the validation group to discriminate malignant from benign lesions. The optimal cut-off value was defined as that maximizing the Youden index. Bar diagrams were plotted to clearly display the discrimination performance of the radiomics score.

#### Calibration

A calibration (i.e., agreement between the observed outcome frequencies and predicted probabilities) curve was plotted to explore the predictive accuracy of the nomogram^16^.

#### Clinical usefulness

Decision curve analysis (DCA) was conducted to determine the clinical usefulness of the nomogram by quantifying the net benefits at different threshold probabilities in the validation group^17^.

The above development and validation methods of the nomogram mainly refer to our previous report^14^.

### Statistical analysis

The details of the statistical analysis mainly refer to our previous report^14^. SPSS 22.0 (Chicago, IL) and R software (version 3.4.1) were used to perform the statistical analysis. The χ² test was used to compare categorical variables. Student’s *t*-test was used to compare continuous variables with a normal distribution. The Mann-Whitney U test was used to compare continuous variables with an abnormal or unknown distribution. The reported statistical significance levels were all two-sided, and *P* values of less than 0.05 were considered statistically significant.

R software was used to develop and assess the nomogram. The “glmnet” package was used for LASSO regression. The “glm” function was used for the univariate and multivariate logistic regression analyses. The “Hmisc” package was used to plot the nomogram. The “pROC” package was used to plot the ROC curves and measure the AUCs, which were compared with DeLong’s test^18,19^. The “Optimal Cut points” package was used for ROC analysis to determine optimal cut-off value. The “ggplot2” package was used to plot bar diagrams. The “CalibrationCurves” package was used for the calibration curves. The “DecisionCurve” package was used to perform DCA.

## Results

### Basic information

Table 1 shows the basic information of the research population. Breast malignancies occurred in 32.2% (68/211) and 33.7% (35/104) of the patients in the training and validation groups, respectively. No significant difference was detected between the two groups for the presence of malignancy (*P* = 0.800). In addition, there were no significant differences between the two groups in the distribution of patient age (*P* = 0.324) or largest lesion diameter (*P* = 0.660). The results showed that there were no significant differences in the baseline characteristics between the two groups. Additional details of the malignant and benign lesions evaluated from the two groups are displayed in Table 2.

**Table 1:** Basic information in the training and validation groups.

	Training (n = 211)	Validation (n = 104)	*P*-value
Age (years)			0.324
>50	34 (16.1%)	24 (23.1%)	
41–50	129 (61.2%)	58 (55.7%)	
≤40	48 (22.7%)	22 (21.2%)	
Diameter (cm)^*^			0.660
>3.0	14 (6.6%)	9 (8.7%)	
2.1–3.0	36 (17.1%)	14 (13.5%)	
1.1–2.0	97 (46.0%)	53 (51.0%)	
≤1.0	64 (30.3%)	28 (26.8%)	
BI-RADS			0.039
4A	153 (72.5%)	71 (68.3%)	
4B	35 (16.6%)	10 (9.6%)	
4C	19 (9.0%)	19 (18.3%)	
5	4 (1.9%)	4 (3.8%)	
Radiomics score^¶^	−0.91 [−1.38, −0.39]	−0.72 [−1.44, −0.21]	0.501
Lesion pathology			0.800
Benign	143 (67.8%)	69 (66.3%)	
Malignant	68 (32.2%)	35 (33.7%)	

^*^Largest diameter of the target lesion.

^¶^Data in parentheses represent interquartile ranges.

**Table 2:** Basic information between the malignant and benign lesions in the two groups.

	Training group (n = 211)		*P*-value	Validation group (n = 104)		*P*-value
	Malignant (n = 68)	Benign (n = 143)		Malignant (n = 35)	Benign (n = 69)	
Age (years)			0.331			0.003
>50	14 (20.6%)	20 (14.0%)		15 (42.9%)	9 (13.0%)	
41–50	37 (54.4%)	92 (64.3%)		14 (40.0%)	44 (63.8%)	
≤40	17 (25.0%)	31 (21.7%)		6 (17.1%)	16 (23.2%)	
Diameter (cm)^*^			<0.001			<0.001
>3.0	10 (14.7%)	4 (2.8%)		7 (20.0%)	2 (2.9%)	
2.1–3.0	19 (27.9%)	17 (11.8%)		8 (22.9%)	6 (8.7%)	
1.1–2.0	30 (44.1%)	67 (46.9%)		18 (51.4%)	35 (50.7%)	
≤1.0	9 (13.2%)	55 (38.5%)		2 (5.7%)	26 (37.7%)	
BI-RADS			<0.001			<0.001
4A	24 (35.3%)	129 (90.2%)		8 (22.9%)	63 (91.3%)	
4B	21 (30.9%)	14 (9.8%)		5 (14.3%)	5 (7.3%)	
4C	19 (27.9%)	0		18 (51.4%)	1 (1.4%)	
5	4 (5.9%)	0		4 (11.4%)	0	
Radiomics score^¶^	−0.33 [−0.74, 0.15]	−1.13 [−1.55, −0.75]	<0.001	−0.05 [−0.49, 0.38]	−1.22 [−1.57, −0.55]	<0.001

^*^Largest diameter of the target lesion.

^¶^Data in parentheses represent interquartile ranges.

### Radiomics score

**Figure 3:**
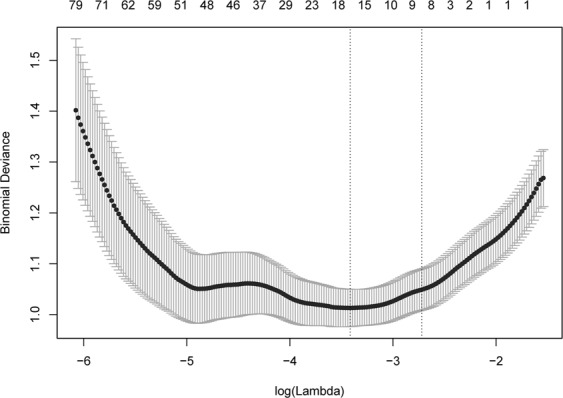
Radiomics feature selection using the least absolute shrinkage and selection operator (LASSO) regression in the training group. The 10-fold cross-validation (CV) process was repeated 50 times to generate the optimal penalization coefficient lambda (λ) in the LASSO model. The value of λ that gave the minimum average binomial deviance was used to select features. Dotted vertical lines were drawn at the optimal values by using the minimum criteria and the 1 standard error of the minimum criteria (the 1-SE criteria). A λ value of 0.066 with log (λ), −2.72 was chosen (the 1-SE criteria) according to the 10-fold CV, where optimal λ resulted in 9 nonzero coefficients.

The intra-observer reproducibility of radiomics feature extraction was good, with ICC values ranging from 0.728 to 0.934. Thus, all statistical analyses are based on the results of the first feature extraction. Based on the training group, 1,044 radiomics features were shrunk to 9 potential predictors by the LASSO regression model (Fig. 3). The 9 features were involved in the radiomics score formula as follows:

$$\begin{array}{rcl}{\rm{Radiomics}}\,{\rm{score}} & = & 2.968901\times {10}^{-4}\times {\rm{Variance}}\\  &  & +\,1.990286\times {10}^{-6}\times {\rm{Relative}}{\rm{Deviation}}\\  &  & -\,9.358726\times {10}^{-3}\times {\rm{Uniformity}}+1.643960\times {10}^{-6}\\  &  & \times \,{\rm{Cluster}}{{\rm{Shade}}}_{-}{\rm{angle}}\,{135}_{-}{\rm{offset}}3+5.166020\times {10}^{-4}\\  &  & \times {\rm{Run}}{\rm{Length}}{{\rm{Nonuniformity}}}_{-}{\rm{All}}{{\rm{Direction}}}_{-}{\rm{offset}}\,{8}_{-}{\rm{SD}}\\  &  & -\,2.703235\times {10}^{-6}\\  &  & \times \,{\rm{Long}}{\rm{Run}}{\rm{High}}{\rm{Grey}}{\rm{Level}}{{\rm{Emphasis}}}_{-}{\rm{All}}{{\rm{Direction}}}_{-}{\rm{offset}}\,{9}_{-}{\rm{SD}}\\  &  & -\,6.461807\times {\rm{Sphericity}}\\  &  & -\,5.195270\times {10}^{-3}\times {\rm{Compactness}}\,1\\  &  & +\,0.133998\times {\rm{Spherical}}{\rm{Disproportion}}\\  &  & -\,1.712025\end{array}$$

The definitions and value ranges of these 9 features are listed in the additional file (Appendix A2 and A3). This formula was used to calculate the radiomics score of each lesion in both groups. There was no significant difference between the training and validation groups in the distribution of the radiomics score (Table 1, *P* = 0.501). Malignant lesions had significantly higher scores than benign lesions in both groups (Table 2, both *P* < 0.001).

**Figure 4:**
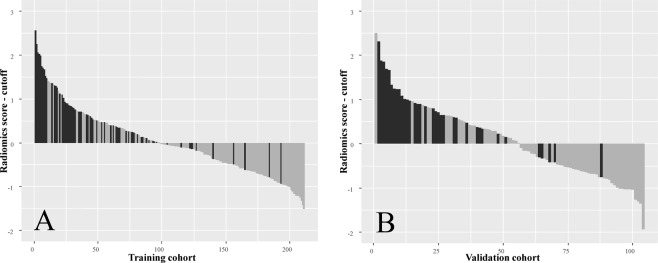
Bar diagrams in the training (**A**) and validation (**B**) groups using the optimal cut-off value of −0.8531 for the radiomics score. The y-axis refers to the radiomics score minus the optimal cut-off value (i.e., radiomics score +0.8531). Up and down bars refer to the predicted malignant and benign lesions, respectively. Black and grey bars refer to actual malignant and benign lesions, respectively.

The optimal cut-off value for the radiomics score for discriminating malignant from benign lesion was −0.8531 in the training group. We used this cut-off value to plot radiomics score bar diagrams in the training (Fig. 4A) and validation (Fig. 4B) groups. The bar diagrams demonstrated the good discrimination performance of the radiomics score.

### Development of the nomogram

**Figure 5:**
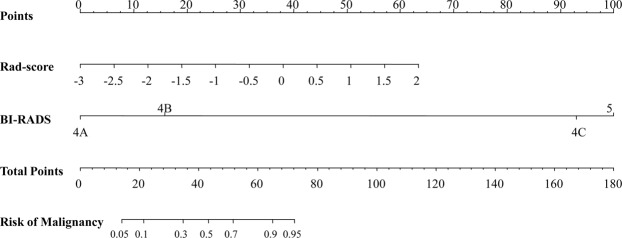
Nomogram with the radiomics score (Rad-score) and BI-RADS category incorporated.

Table 3 displays the results of univariate and multivariate analyses for breast malignancy in the training group. The radiomics score and BI-RADS category were demonstrated to be independent predictors of breast malignancy (both *P* < 0.001). Therefore, the nomogram was built with the BI-RADS category and radiomics score (Fig. 5).

**Table 3:** Results of the univariate and multivariate analyses based on the training group.

	Univariate analysis		Multivariate analysis	
	OR (95% CI)	*P*-value	OR (95% CI)	*P*-value
Age (years)				
>50	Ref.		—	—
41–50	0.57 (0.26, 1.27)	0.165	—	—
≤40	0.78 (0.32, 1.94)	0.596	—	—
Diameter (cm)^*^				
>3.0	Ref.		Ref.	
2.1–3.0	0.45 (0.11, 1.61)	0.236	2.24 (0.27, 21.54)	0.458
1.1–2.0	0.18 (0.05, 0.58)	0.006	3.57 (0.44, 35.76)	0.246
≤1.0	0.07 (0.02, 0.24)	<0.001	3.86 (0.35, 52.47)	0.284
BI-RADS				
4A	Ref.		Ref.	
4B	8.06 (3.65, 18.41)	<0.001	4.66 (1.88, 11.84)	<0.001
4C	NA	0.989	NA	0.989
5	NA	0.995	NA	0.995
Radiomics score	7.04 (4.04, 13.35)	<0.001	4.87 (2.02, 12.78)	<0.001

^*^Largest diameter of the target lesion.

NA, values were not available.

### Validation of the nomogram

#### Discrimination

**Figure 6:**
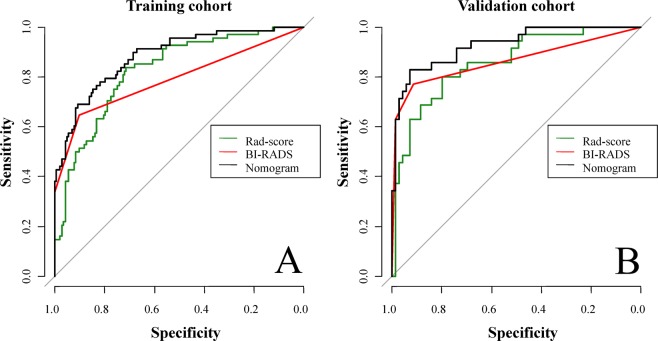
Receiver operating characteristic (ROC) curves of the radiomics score (green lines), BI-RADS category (red lines) and nomogram (black lines) in the training (**A**) and validation (**B**) groups, respectively.

Table 4 displays the performance of the nomogram, BI-RADS category and radiomics score for discriminating between malignant and benign lesions. The results of AUCs for the nomogram, BI-RADS category and radiomics score were 0.928 [95% confidence interval (CI): 0.876, 0.980], 0.864 (95% CI: 0.787, 0.941) and 0.857 (95% CI: 0.780, 0.933), respectively, in the validation group and 0.883 (95% CI: 0.835, 0.932), 0.791 (95% CI: 0.729, 0.854) and 0.825 (95% CI: 0.767, 0.884), respectively, in the training group. The BI-RADS category and radiomics score showed similar discrimination in both groups (*P* = 0.357 and *P* = 0.882 for the training and validation groups, respectively). The nomogram incorporating the BI-RADS category and radiomics score showed significantly better discrimination than the radiomics score (*P* = 0.003) or BI-RADS category (*P* < 0.001) in the training group. These significant differences were demonstrated in the validation group (nomogram *vs*. radiomics score, *P* = 0.029; nomogram *vs*. BI-RADS category, *P* = 0.011). Figure 6 shows the ROC curves of the nomogram, BI-RADS category and radiomics score in both groups. The nomogram displayed the best discrimination performance.

**Table 4:** AUCs of the radiomics score, BI-RADS and nomogram.

	Training group (n = 211)		Validation group (n = 104)	
	AUC (95% CI)	*P*-value	AUC (95% CI)	*P*-value
Nomogram	0.883 (0.835, 0.932)		0.928 (0.876, 0.980)	
BI-RADS	0.791 (0.729, 0.854)		0.864 (0.787, 0.941)	
Radiomics score	0.825 (0.767, 0.884)		0.857 (0.780, 0.933)	
Nomogram *vs*. BI-RADS		<0.001		0.011
Nomogram *vs*. Radiomics score		0.003		0.029
BI-RADS *vs*. Radiomics score		0.357		0.882

#### Calibration

**Figure 7:**
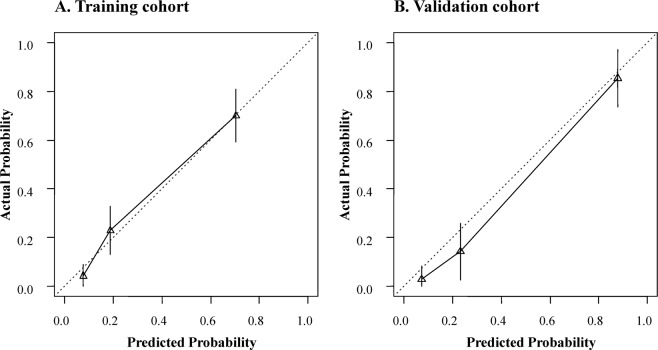
Calibration curves for the nomogram in training (**A**) and validation groups (**B**), respectively.

The calibration curves of the nomogram applied in the training and validation groups are shown in Fig. 7a,b, respectively. The nomogram showed good agreement for detecting breast malignancy between prediction and histopathologic confirmation.

#### Clinical usefulness

**Figure 8:**
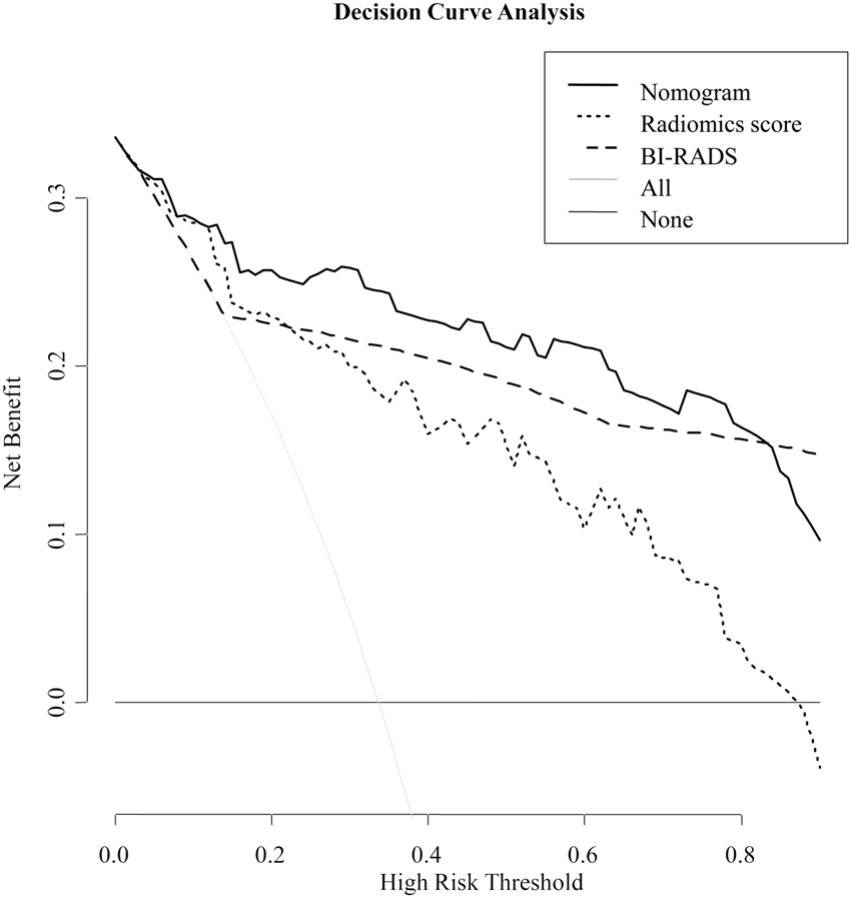
Decision curve analysis (DCA) derived from the validation group. The y-axis measures the net benefit. The net benefit is determined by calculating the difference between the expected benefit and the expected harm associated with each proposed model [Net benefit = true positive rate − (false positive rate × weighting factor), weighting factor = Threshold probability/(1-threshold probability)]. The grey line represents the assumption that all lesions were malignant (the treat-all scheme). The black line represents the assumption that all lesions were benign (the treat-none scheme). If the threshold probability was more than 5%, using the nomogram to predict malignancy added more benefit than either the treat-all scheme or the treat-none scheme (dark black line).

DCA was used to assess the clinical usefulness of the nomogram, BI-RADS category and radiomics score in the validation group (Fig. 8). If the threshold probability was more than 5%, using the nomogram to predict malignancy added more benefit than either the treat-all scheme (assuming that all lesions were malignant) or the treat-none scheme (assuming that all lesions were benign). In addition, using the nomogram to predict malignancy added more benefit than either using only the radiomics score or using only the BI-RADS.

## Discussion

In the present study, a radiomics score was developed to predict malignancy in breast lesions classified as BI-RADS US category 4 or 5. The radiomics score was independently associated with breast malignancy. A nomogram incorporating the radiomics score and BI-RADS category showed strong discrimination performance of malignant and benign lesions. The calibration curve showed that the predicted and actual probability of breast malignancy were in good agreement. DCA demonstrated good clinical usefulness of the nomogram.

Radiomics is a rapidly developing computer-aided technology that converts medical imaging information into a series of data through computer algorithms^5,6^. Previous studies have shown that image microscopic characteristics are closely related to tumour microstructure and biological behaviour^20–23^. Radiomics features reflect the texture features of tumours, which are important biomarkers of tumour heterogeneity. However, the association between biological behaviour and radiomics features is still complex^24^. When biomarkers are selected from thousands of radiomics features, it is difficult to clearly clarify the relationship between radiomics features and biological behaviour. An effective method is to use radiomics techniques to establish multi-feature parameters for the estimation of results^25,26^. In our previous study, we built a radiomics score consisting of 19 selected radiomics features to predict malignancy in thyroid nodules^27^. The radiomics score was demonstrated to have good discrimination between malignant and benign thyroid nodules, with AUCs of 0.921 and 0.931 in the training and validation groups, respectively. In the present study, radiomics features of breast lesions were extracted from greyscale US images, and 9 significant features were selected to build the radiomics score. The radiomics score was an independent predictor for breast malignancy and showed good performance for discriminating malignant from benign lesions, with AUC of 0.825 and 0.857 in the training and validation groups, respectively.

The radiomics score consisted of 9 radiomics features, including 3 histogram parameters (Variance, RelativeDeviation, Uniformity), 1 texture parameter (ClusterShade_angle135_offset3), 2 grey level run-length matrix (RLM) parameters (RunLengthNonuniformity_AllDirection_offset8_SD, LongRunHighGreyLevelEmphasis_AllDirection_offset9_SD) and 3 form factor parameters (Sphericity, Compactness1, SphericalDisproportion) (Appendix A2). Since the values of features extracted from the A.K. software were not standardized, and we did not standardize the data during the analysis, all values of features are expressed using their own scales. Thus, the relative contribution of different features to the radiomics score cannot be simply evaluated by the coefficients. After combining the value range (Appendix A3) and the coefficient of each feature, the Sphericity and SphericalDisproportion seemed to contribute most to the radiomics score, followed by the ClusterShade_angle135_offset3. Therefore, the radiomics score may be most closely related to the shape of the region. The radiomic score might represent tumour shape and border irregularities more than tumour texture.

In present study, the false positive rates of lesions classified as BI-RADS 4A, 4B, 4C and 5 were 84.3% (129/153), 40.0% (14/35), 0 (0/19) and 0 (0/4), respectively, in the training group and 88.7% (63/71), 50.0% (5/10), 5.3% (1/19) and 0 (0/4), respectively, in the validation group. Although the radiomics score showed good performance for discriminating malignant from benign lesions, false positive results were still inevitable. Use of the radiomics score resulted in 42.3% (41/97) and 46.4% (26/56) false positives (according to the optimal cut-off value of −0.8531) in the training and validation groups, respectively, which were similar to the rates found for lesions classified as BI-RADS 4B. These results indicated that nearly half of lesions were classified as suspicious malignancy according to the radiomics score but were finally shown to be benign after biopsy. Therefore, a further biopsy is still needed when a lesion has a high radiomics score. However, we also noticed that as the radiomics score increased, fewer false positives occurred (Fig. 4). This potential correlation may be clinically useful. More research studies are needed to explore the relationship between radiomics and false positives.

Nomograms have been widely used to predict medical prognosis and outcomes by combining multiple risk factors. *Huang et al*. incorporated a radiomics signature and clinical risk factors into a nomogram^11^. The nomogram performed better for predicting disease-free survival in early-stage non-small cell lung cancer than clinical risk factors used alone^11^. In the present study, the radiomics score and BI-RADS category were independent predictors of breast malignancy. A nomogram was then developed by incorporating the above two variables. The nomogram showed good performance for malignancy prediction (AUC of 0.928), which was significantly higher than that of the radiomics score (*P* = 0.029) or BI-RADS category (*P* = 0.011) in the validation group (Table 4). However, examination of the nomogram suggested that the radiomic score does not have any added value for lesions with a BI-RADS category of 4C or 5, given that the BI-RADS category already yields more than 90 points, indicating a greater than 95% probability of malignancy. In the ACR BI-RADS, subcategory 4C represents a 50–95% likelihood of malignancy^4^, which is less than the malignant probability of BI-RADS 4C lesions in the present study. The reason for this difference may be that the radiologist (**W.L**., with over 10 years of experience in breast US examination) was experienced in discriminating breast US features.

The calibration curve of the nomogram is used to assess the agreement between the predicted and actual malignant probability^16^. In our study, the nomogram showed high accuracy for individual predictions in the validation and training groups (Fig. 7). DCA was used to assess whether the nomogram led to improved individual benefit. This method is based on a clinical outcome analysis of threshold probabilities to calculate the net benefit of the population. Net benefit is defined as the proportion of true positives minus the proportion of false positives weighted by the relative harm of false-positive and false-negative results^28^. Notably, DCA showed that the nomogram added more benefit for predicting breast malignancy than either the treat-all scheme (assuming all lesions were malignant) or the treat-none scheme (assuming all lesions were benign).

There were several limitations in the present study. First, in a retrospective study, bias is inevitable. Prospective studies are needed to control for confounding variables. Second, the present study was a single-centre research study. In our study, although the performance of the nomogram has been evaluated by an independent validation cohort, additional validation at other centres will be necessary to assess the reliability of this prediction model. Third, in our study, only one radiologist (**W.L**., with over 10 years of experience in breast US examination) performed the US examinations, assigned BI-RADS categories for breast lesions, and delineated ROIs for extracting radiomics features. This may limit the application of the radiomics score for breast US examinations performed by other radiologists who are likely to have different results. However, our results demonstrated that the radiomics score showed similar discrimination performance to BI-RADS classification, and the nomogram showed better performance than the radiomics score or BI-RADS category. These results demonstrated that incorporating radiomics with BI-RADS category could improve the predictive performance for identifying breast malignancy, which can likely be reproduced by other radiologists. In the next study, we will investigate the efficiency of radiomics in breast US performed by different radiologists with different levels of experience.

## Conclusions

In our study, we established an index called the radiomics score based on US images of patients with breast lesions assessed as BI-RADS US category 4 or 5. The radiomics score may be considered a potential biomarker for predicting breast malignancy. The nomogram, which combined the radiomics score and BI-RADS category, demonstrated good discrimination performance between malignant and benign lesions as well as good calibration and clinical usefulness. Therefore, the nomogram has potential application value for breast cancer prediction in breast lesions classified as BI-RADS US category 4 or 5.

## Supplementary information

Additional file
