# Supplementary material for: Predicting Breast Cancer in Breast Imaging Reporting and Data System (BI-RADS) Ultrasound Category 4 or 5 Lesions: A Nomogram Combining Radiomics and BI-RADS
Source: Sci Rep. 2019 Aug 15;9:11921. doi: 10.1038/s41598-019-48488-4 (PMC6695380; doi:10.1038/s41598-019-48488-4)
Supplement: Supplementary file 1 — Additional file [file 41598_2019_48488_MOESM1_ESM.docx]

**Predicting Breast Cancer in Breast Imaging Reporting and Data System (BI-RADS) Ultrasound Category 4 or 5 Lesions: A Nomogram Combining Radiomics and BI-RADS**

Wei-quan Luo, Qing-xiu Huang, Xiao-wen Huang, Hang-tong Hu, Fu-qiang Zeng, Wei Wang

**Appendix A1: Process of building the radiomics score formula**

First, a total of 315 grayscale US images (211 for the training group and 104 for the validation group) with the largest long axis cross-section of all target lesions were exported from the US machine and imported into the A.K. software (Artificial Intelligence Kit, version 1.1, GE Healthcare, Little Chalfont, UK). Then, the radiologist delineated a region of interest (ROI) around the margin of each target lesion. The software automatically extracted the radiomics features from the ROI. In total, 1,044 features were ultimately extracted from each ROI. These 1,044 features consisted of four categories of features: gray-level histogram, transformed-matrix texture, wavelet-transformed texture and filter-transformed texture. With the histogram feature, we extracted the texture feature parameters and developed a quantitative or qualitative description of the texture based on the gray values of the images. The transformed-matrix texture extracted high-level information from the ROI by a series of matrix transformations, such as gray-level co-occurrence matrices (GLCMs) and run-length matrices (RLMs). With the wavelet-transformed texture, we analysed the characteristics of the ROI with different levels of resolution. With the filter-transformed texture, we obtained a series of target features with different types of filters, such as log transformation and Gaussian transformation.

Second, features associated with lesion malignancy in the training group were selected from these 1,044 features. Because of the large number of image features and the relatively small group size, we used least absolute shrinkage and selection operator (LASSO) regression to perform the reduction and selection of features. LASSO regression, a type of multivariate linear regression, shrinks the coefficients towards zero by setting a constraint on the sum of the absolute standardized coefficients. Shrinkage estimates made with the LASSO method simultaneously remove non-informative variables and estimate the odds ratios (ORs) for the informative features. This provided an important method of adjusting for model overfitting and preventing extreme predictions. The method uses a lambda (λ) penalty to shrink coefficients. The penalty parameter λ controls the amount of shrinkage such that the larger the value of λ is, the fewer the features that are selected. The 10-fold cross-validation process was repeated 50 times to generate the optimal value of the LASSO penalty parameter λ. The value of λ that provided the minimum average binomial deviance for the training group was used to select features.

Finally, the radiomics score formula was built using the selected features. Multivariate logistic regression analyses were used to generate the coefficients of all selected features from LASSO regression. Based on a linear combination of the selected features weighted by their coefficients, we calculated a radiomics score for each lesion using the following formula: radiomics score $= \sum_{i=1}^{n} Xi$, where *X* is the selected features, and *n* is the number of selected features.

**Appendix A2: Definition of the 9 features that are involved in the Radiomics score formula**

**1. Variance**: histogram parameter.

Histogram parameters are concerned with properties of individual pixels. They describe the distribution of voxel intensities within the image through commonly used and basic metrics.

Variance is the average of the squared differences from the Mean.

$$\boldsymbol{variance}\boldsymbol{=}\frac{\boldsymbol{1}}{\boldsymbol{N-1}}\sum_{\boldsymbol{i=1}}^{\boldsymbol{N}} {\boldsymbol{(}\boldsymbol{X}\left( \boldsymbol{i} \right)\boldsymbol{-} \bar{\boldsymbol{X}}\boldsymbol{)}}^{\boldsymbol{2}}$$

where $\bar{X}$ is the mean of *X*.

**2. RelativeDeviation**: histogram parameter.

Let $\bar{X}$ denote the mean of a set of quantities $X_{i}$, then the relative deviation is defined by:

$$\frac{\boldsymbol{\Delta}\boldsymbol{X}_{\boldsymbol{i}}}{\overline{\boldsymbol{X}}}\boldsymbol{=}\frac{\left| \boldsymbol{X}_{\boldsymbol{i}}\boldsymbol{-}\overline{\boldsymbol{X}} \right|}{\overline{\boldsymbol{X}}}$$

**3. Uniformity**: histogram parameter.

$$\boldsymbol{uniformity}\boldsymbol{=}\sum_{\boldsymbol{i=1}}^{\boldsymbol{N}} {\boldsymbol{P}\left( \boldsymbol{i} \right)}^{\boldsymbol{2}}$$

**4.** **ClusterShade_angle135_offset3**: texture parameter.

Cluster analysis or clustering is the task of grouping a set of objects in such a way that objects in the same group (cluster) are more similar (in some sense or another) to each other than to those in other groups (clusters). It is a common technique for statistical data analysis. Cluster Shade in clustered shading, we group similar view samples according to their position and, optionally, normal into clusters.

$$\sum_{\boldsymbol{i}\boldsymbol{,}\boldsymbol{j}} \left[ \left( \boldsymbol{i}\boldsymbol{-}\boldsymbol{\mu} \right)\boldsymbol{+}\left( \boldsymbol{j}\boldsymbol{-}\boldsymbol{\mu} \right) \right]^{\boldsymbol{3}}\boldsymbol{g}\left( \boldsymbol{i}\boldsymbol{,}\boldsymbol{j} \right)$$

where *μ* is the mean of the row (or column, due to symmetry) sums.

**g* is a Grey level co-occurrence matrix (GLCM) that represents the joint probability of certain sets of pixels having certain grey-level values. It calculates how many times a pixel with grey-level *i* occurs jointly with another pixel having a grey value *j*.

In A.K. software, there are 36 parameters related to Cluster analysis, which include the ClusterShade_angle135_offset3.

**5. RunLengthNonuniformity_AllDirection_offset8_SD**: RLM parameter.

The grey level run-length matrix (RLM) $P_{r}\left( i,j | \theta\right)$is defined as the numbers of runs with pixels of gray level *i* and run length *j* for a given direction *θ*. RLMs are generated for each sample image segment having directions (0°,45°,90° & 135°), then the following ten statistical features were derived: short run emphasis, long run emphasis, grey level non-uniformity, run length non-uniformity, Low Grey Level Run Emphasis, High Grey Level Run Emphasis, Short Run Low Grey Level Emphasis, Short Run High Grey Level Emphasis, Long Run Low Grey Level Emphasis and Long Run High Grey Level Emphasis.

The formula of Run Length Non-uniformity is as follows:

$$\boldsymbol{RLN(\theta)}\boldsymbol{=}\frac{\boldsymbol{1}}{\boldsymbol{n}_{\boldsymbol{r}}}\sum_{\boldsymbol{j=i}}^{\boldsymbol{N}} \boldsymbol{(}\sum_{\boldsymbol{i=1}}^{\boldsymbol{M}} \boldsymbol{p}{\boldsymbol{(}\boldsymbol{i,j,\theta}\boldsymbol{))}}^{\boldsymbol{2}}$$

In A.K. software, there are 18 Run Length Non-uniformity parameters, which include the RunLengthNonuniformity_AllDirection_offset8_SD.

**6. LongRunHighGreyLevelEmphasis_AllDirection_offset9_SD**: RLM parameter.

The formula of Long Run High Grey Level Emphasis is as follows:

$$\boldsymbol{LRHGE(\theta)}\boldsymbol{=}\frac{\boldsymbol{1}}{\boldsymbol{n}_{\boldsymbol{r}}}\sum_{\boldsymbol{j=i}}^{\boldsymbol{N}} \sum_{\boldsymbol{i=1}}^{\boldsymbol{M}} \boldsymbol{p}{\boldsymbol{(}\boldsymbol{i,j,\theta}\boldsymbol{)i}}^{\boldsymbol{2}}\boldsymbol{j}^{\boldsymbol{2}}$$

Where *n_r_* is the total number of runs.

In A.K. software, there are 18 Long Run High Grey Level Emphasis parameters, which include the LongRunHighGreyLevelEmphasis_AllDirection_offset9_SD.

**7.** **Sphericity**: form factor parameter.

Form factor parameters include descriptors of the three-dimensional size and shape of the tumour region. Let in the following definitions *V* denote the volume and *A* the surface area of the volume of interest.

$$\boldsymbol{sphericity}\boldsymbol{=}\frac{\boldsymbol{\pi}^{\frac{\boldsymbol{1}}{\boldsymbol{3}}}{\boldsymbol{(6}\boldsymbol{V)}}^{\frac{\boldsymbol{2}}{\boldsymbol{3}}}}{\boldsymbol{A}}$$

**8. Compactness1**: form factor parameter.

$$\boldsymbol{compactness}\boldsymbol{1}\boldsymbol{=}\frac{\boldsymbol{V}}{\sqrt{\boldsymbol{\pi}}\boldsymbol{A}^{\frac{\boldsymbol{2}}{\boldsymbol{3}}}}$$

**9. SphericalDisproportion**: form factor parameter.

$$\boldsymbol{spherical disproportion}\boldsymbol{=}\frac{\boldsymbol{A}}{\boldsymbol{4}\boldsymbol{\pi}\boldsymbol{R}^{\boldsymbol{2}}}$$

Where *R* is the radius of a sphere with the same volume as the tumour.

**Appendix A3: value ranges of the 9 features that are involved in the Radiomics score formula**

**Table A1 Distribution of the 9 selected features between the malignant and benign lesions in two groups**

|  | Training group (n=211) | | Validation group (n=104) | |
| --- | --- | --- | --- | --- |
|  | Malignant | Benign | Malignant | Benign |
| Variance | 548.88 [324.51, 724.16] | 450.70 [326.65, 586.88] | 485.41 [300.04, 679.02] | 461.90 [306.96, 570.42] |
| RelativeDeviation | 43207.75 [18404.33, 97829.85] | 16038.80 [8382.83, 29476.60] | 67836.30 [25311.60, 90458.60] | 15729.30 [8969.45, 29201.55] |
| Uniformity | 0.61 [0.55, 0.66] | 0.68 [0.61, 0.72] | 0.60 [0.52, 0.68] | 0.66 [0.60, 0.70] |
| ClusterShade_angle135_offset3 | 198353.00 [145891.75, 248135.50] | 132344.00 [73361.00, 214750,00] | 229001.00 [112906.00, 310850.00] | 163335.00 [72109.40, 254221.00] |
| RunLengthNonuniformity_AllDirection_offset8_SD | 119.33  [58.85, 232.35] | 55.21 [24.39, 91.49] | 144.99 [81.00, 325.82] | 44.37 [24.84, 76.73] |
| LongRunHighGreyLevelEmphasis_AllDirection_offset9_SD | 272.68 [198.10, 409.67] | 365.92 [249.12, 577.92] | 273.92 [190.98, 399.67] | 359.40 [241.87, 490.00] |
| Sphericity | 0.11 [0.08, 0.12] | 0.14 [0.11, 0.16] | 0.09 [0.08, 0.11] | 0.14 [0.11, 0.16] |
| Compactness1 | 0.20 [0.18,0. 28] | 0.31 [0.26, 0.45] | 0.20 [0.16, 0.28] | 0.36 [0.28, 0.48] |
| SphericalDisproportion | 9.43 [8.41, 12.22] | 7.39 [6.08, 8.84] | 10.53 [8.79, 12.59] | 7.15 [6.14, 8.79] |

Data in parentheses represents interquartile ranges
